# Clinical evaluation and finite element analysis of bone cement-augmented anterolateral screw fixation versus percutaneous bilateral pedicle screw fixation co-applied with oblique lumbar interbody fusion for single-level lumbar degenerative diseases with osteoporosis

**DOI:** 10.3389/fbioe.2025.1571849

**Published:** 2025-06-10

**Authors:** Xiaoping Mu, Xiaodong Wei, Jiahong Nong, Huabao Ye, Zhuhai Li, Minke Wei, Jianxun Wei

**Affiliations:** ^1^ Department of Spine Surgery, The People’s Hospital of Guangxi Zhuang Autonomous Region, Nanning, China; ^2^ Guangxi Academy of Medical Sciences, Nanning, China; ^3^ Graduate School, Youjiang Medical University for Nationalities, Baise, China; ^4^ Department of Orthopaedics, People’s Hospital of Luchuan, Yulin, China

**Keywords:** intervertebral disc degeneration, spinal stenosis, osteoporosis, oblique lateral interbody fusion, finite element model, bone cement-augmented screw

## Abstract

**Background:**

As the population ages, there is an increasing trend in patients with lumbar degenerative diseases (LDD) complicated by osteoporosis seeking lumbar fusion surgery. However, standardized strategies for minimally invasive surgical procedures among these populations still need improvement in clinical practice.

**Purpose:**

This study was to integrate clinical and biomechanical approaches to investigate and demonstrate the effectiveness of oblique lateral interbody fusion combined with bone cement-augmented anterolateral screw (OLIF-BCAAS) in such patients.

**Study Design:**

A single-center, retrospective case-controlled clinical study and finite element model (FEM) analysis.

**Methods:**

A single-center, retrospective case-controlled clinical study and finite element model (FEM) analysis were conducted. 48 cases were enrolled in the clinical study, then assigned to either OLIF-BCAAS or OLIF combined with posterior internal fixation with pedicle screws (OLIF-PIFPS). Clinical outcomes and radiological parameters were statistically analyzed. The FE models of intact lumbar spine, OLIF-BCAAS, and OLIF-PIFPS were constructed based on computed tomography (CT) scans of a healthy male. These FE models were analyzed under different loading conditions.

**Results:**

There were significant differences in the surgical time, blood loss, and lower back score within 1 year postoperatively between the two groups (p < 0.05). Moreover, both OLIF surgical techniques showed significant improvements in disc height (DH) postoperatively; however, the reduction in DH at postoperative 12 months was more pronounced in the OLIF-PIFPS group than in the OLIF-BCAAS group (p < 0.05). Five cases (5/23, 21.74%) of cage subsidence (CS) were detected in the OLIF-BCAAS group, with 4 out of 23 cases (17.39%) considered as mild CS. In contrast, the amount of CS was 12 cases (12/25, 48%) in the OLIF-PIFPS group, which included 3 cases of severe CS. However, there was a trend towards statistical difference in CS between the two groups (p = 0.057). The FEM analysis showed significant reductions in the local range of motion and L3 maximum displacement with respect to L4 under six motion patterns in the two OLIF surgical models. Moreover, stress on the endplate and cage in the OLIF-BCAAS model was higher than that in the OLIF-PIFPS model; however, stress on the supplemental fixation devices was significantly lower than that observed in the OLIF-PIFPS model.

**Conclusion:**

Both OLIF surgical techniques for treating LDD with osteoporosis can achieve favorable clinical outcomes. However, OLIF-BCAAS exhibits more significant advantages over OLIF-PIFPS by maximizing the benefits of minimally invasive surgery. Moreover, OLIF-BCAAS is associated with lower postoperative back pain and a reduced incidence of postoperative CS.

## Introduction

For patients experiencing suboptimal outcomes from conservative treatments, lumbar fusion stands out as the conventional approach in addressing lumbar degenerative diseases (LDD) combined with lumbar instability (Tsagkaris et al., 2023; Machado et al., 2015). According to the epidemiological report (Maurer et al., 2022), LDD predominantly manifests in the middle-aged and elderly demographic. Within this cohort, there is often a correlation with low bone mass or osteoporosis. Decreased bone strength emerges as a pivotal factor influencing the stability of pedicle screws in the vertebrae body (Li W. et al., 2023), which can lead to postoperative screw loosening, cage subsidence (CS), or even non-union, significantly compromising postoperative effectiveness (Tsagkaris et al., 2023; Kim D. H. et al., 2020). Previous studies (Li W. et al., 2023; Kim D. H. et al., 2020; Li Y. D. et al., 2023) further substantiate that the decline in bone density substantially augments the likelihood of screw loosening and non-union following lumbar fusion. Moreover, anesthesia and lumbar surgery pose inherent challenges for the elderly patients. Therefore, the quest for a more minimally invasive lumbar fusion coupled with a robust internal fixation procedure undoubtedly represents the crux of resolving this inherent contradiction.

Posterior lumbar interbody fusion with internal fixation remains the primary procedure for LDD combined with osteoporosis. Nevertheless, this technique is always associated with significant disruption of the posterior lumbar bone and soft tissues, along with frequent neural disturbances (Formby et al., 2016). The extended postoperative recovery makes it less favorable for elderly patients and is misaligned with the prevailing concept of rapid rehabilitation. Oblique lateral interbody fusion (OLIF) has garnered acknowledgment as an efficacious approach for addressing LDD with instability. Since its initial report in 2012 (Silvestre et al., 2012), OLIF has demonstrated advantages attributed to minimal surgical trauma, expedited postoperative recovery, and definitive clinical efficacy. The clinical applications of OLIF have steadily broadened alongside advancements in surgical techniques, positioning it as the evolving mainstream procedure in contemporary clinical practice.

Performing OLIF on patients with concomitant LDD and osteoporosis has been associated with increased postoperative CS (Kotheeranurak et al., 2023; Zhao et al., 2022). However, for patients with a bone density of T-score less than −1.0 standard deviation (SD), an alternative strategy entails executing posterior bilateral pedicle screw fixation after the anterior cage implantation, thereby averting CS (Cai et al., 2021). Nevertheless, this strategy mandates intraoperative adjustments in patient positioning, encompassing lateral and prone orientations. This extends the duration of surgical and anesthesia, and introduces the potential for complications during positional transitions. Moreover, the combined procedure involves two distinct surgical procedures, resulting in significantly greater surgical trauma compared to any singular approach, posing challenges for postoperative recovery, particularly in elderly patients.

In the management of such patients, the clinical application of cement-augmented pedicle screw fixation has become widespread, with its effectiveness validated by previous studies (Elder et al., 2015; Shafiekhani et al., 2023; Tandon et al., 2020). However, this technique still has its drawbacks: 1) The fast setting time of bone cement reduces the surgeon’s operational time; 2) The distribution of bone cement is uncontrollable, which can lead to bone cement leakage (Janssen et al., 2017); 3) The insertion of screws may cause displacement of the bone cement, leading to severe complications such as nerve and vascular damage, or even bone cement embolism (Canyang et al., 2023). Moreover, bone cement implantation syndrome (BCIS) may occasionally occur during surgery or the perioperative period (Hines, 2018). A recent study (Moldovan, 2023) provided a comprehensive analysis of 6 cases of BCIS, along with a thorough narrative review of existing literature on BCIS, which may assist clinicians in the early recognition and effective management of this complication.

The most widely used bone cement material in orthopedic surgery is still polymethyl methacrylate (PMMA). However, PMMA does not possess adhesive properties. Its fixation mechanism primarily relies on the firm mechanical interlocking between the irregular gaps on the bone surface and the implant, making it more like a space filler (Vaishya et al., 2013). Its poor osteoconductivity and the high-temperature issues caused by the exothermic polymerization reaction limit its broad application (Karpiński et al., 2023a). Adding specific additives to PMMA to improve the performance of bone cement has been a hot research topic. Previous studies have analyzed various additives combined with PMMA, such as Glassy carbon (Karpiński et al., 2023a), hydroxyapatite (Karpiński et al., 2023b), and α/β tricalcium phosphate (Karpiński et al., 2023c), and reported significant research findings.

Theoretically, the attainment of effective decompression, fusion, and sturdy fixation for such patients can be successfully completed through a singular oblique anterior approach. This involves the simultaneous placement of cage and anterolateral screws reinforced with bone cement augmentation. Therefore, the purpose of the present study was to compare the clinical and radiological outcomes, and demonstrate the mechanisms performance of bone cement-augmented anterolateral screw (BCAAS) versus percutaneous posterior internal fixation with pedicle screws (PIFPS) co-applied with OLIF in the treatment of single-level LDD with osteoporosis. This study was reported in line with The STROCSS 2024 guideline (Rashid et al., 2024): strengthening the reporting of cohort, cross-sectional, and case-control studies in surgery.

## Materials and methods

The setting of the present work was a single-center, retrospective case-controlled clinical study and basic study of three-dimensional finite element model (FEM) analysis. This study was conducted in strict accordance with the principles of the Helsinki Declaration. We obtained written informed consent from the study participants. Approval for the study protocol was obtained from our institutional ethics committee.

### Part I clinical study

#### Inclusion and exclusion criteria

In accordance with the study purpose, we have established the following inclusion criteria: 1) Adult patients who meet the diagnostic criteria for LDD (including lumbar spinal stenosis, lumbar disc herniation, and I-II° degenerative lumbar spondylolisthesis) combined with lumbar instability (≥3 mm of slippage or an angle ≥11° between two adjacent vertebrae in dynamic lumbar X-rays, or an angle ≥5° between the vertebral endplates on hyperflexion X-rays, or vertebral rotation and imbalance on the anteroposterior X-ray) (Elmose et al., 2023; Orthopaedic Rehabilitation Group of the Orthopaedic Society of the Chinese Medical Association, 2025); 2) Ineffectiveness of conservative treatment for more than 3 months; 3) Dual-energy X-ray absorptiometry (DXA) revealing a bone density of T-score less than −2.5 SD at the surgical lumbar level; 4) Undergoing either single-level OLIF-BCAAS or OLIF-PIFPS during the study period; 5) Demonstration of willingness to participate in the study, provision of informed consent regarding the research content, and the ability to collaborate in assessments and data collection at various follow-up points.

Refinement of the exclusion criteria is outlined as follows: 1) Cases necessitating direct decompression of the spinal canal, such as bony stenosis of the central vertebral canal or prolapsed lumbar disc; 2) Presence of a history of prior lumbar spine surgery; 3) Existence of multiple underlying diseases that make the patient intolerant to surgery or anesthesia; 4) Concurrent manifestation of other conditions, such as lower back pain or functional impairment in lower limb, stemming from factors unrelated to the study, which could potentially impact the assessment of treatment efficacy.

Finally, this study enrolled 48 cases diagnosed with LDD combined with lumbar instability and osteoporosis in our department from January 2020 to July 2023. The experimental group comprised 23 patients who underwent single-level OLIF-BCAAS. Meanwhile, 25 patients who underwent OLIF-PIFPS were allocated to the control group. The same surgical team performed all surgical procedures for the patients included in this study.

#### Surgical techniques

Both cohorts of patients underwent the standard surgical procedure of OLIF in the right lateral decubitus position after the successful administration of general anesthesia. In the experimental group, we identified the sagittal midpoint of the cage and approximately 5–10 mm to the edge of the upper/lower vertebral endplate as the screw insertion point after the implantation of the cage. A Washer was initially inserted into the left side of the vertebral body using a hammer. Subsequently, we used a taper to drill a pilot hole parallel to the vertebral endplate along the axis of the cage until 3–5 mm away from the contralateral cortex bone. Following the injection of approximately 1.5 mL of bone cement through the pilot hole, an anterolateral screw with an appropriate size was inserted into the vertebral body and a connecting rod with the appropriate length was placed. The patient was placed in the prone position after the cage implantation in the controlled group. We performed the standardized procedure of percutaneous bilateral pedicle screw insertion. All the cases included in this study were performed by the same senior spinal surgeon (J.X.W.) with over 10 years of clinical experience.

#### Clinical outcomes and radiological evaluation

Clinical outcomes for each subject were collected by two authors, including: 1) Baseline characteristics: patient’s age (years), sex, body mass index (BMI), bone mass density (BMD), operative level, operative time (min), intraoperative blood loss (mL), and hospital stay (days). 2) Functional outcomes: visual analogue scale (VAS) and Japanese Orthopaedic Association score (JOA) preoperatively, at postoperative 3, 6, and 12 months. Each subject was examined for the lumbar X-ray and computed tomography (CT) scan preoperatively, 1–2 days postoperatively, and again 12 months after surgery. 3) Surgical-related complications: vascular or nerve injuries, cerebrospinal fluid leaks, surgical site infections, loosening/dislocation of internal fixation, and the need for reoperation.

Two authors independently measured the following radiological parameters: 1) Disc height (DH, mm), defined as the vertical distance between the mid-point of the upper and lower endplate on the lumbar X-ray. 2) Cage subsidence (CS), described as a reduction in intervertebral height of more than 2 mm on the lumbar X-ray (mild CS: 2–4 mm, severe CS: >4 mm). 3) Fusion status, we adopted the anterior fusion grade criteria proposed by Bridwell et al. (1995) to evaluate the postoperative intervertebral fusion (fused: grade 1 and 2, unfused: grade 3 and 4) on the lumbar X-rays scan at the last follow-up. This grading system consists of the following categories: Grade I: fused with remodeling and trabeculae; Grade II: graft intact, not fully remodeled and incorporated though, no lucencies; Grade III: graft intact, but a definite lucency at the top or bottom of the graft; IV definitely not fused with resorption of bone graft and with collapse.

#### Statistical analysis

Statistical analysis of this study was performed using SPSS software (Version 22.0. Armonk, NY: IBM Corp.). The statistical description of continuous data was presented as mean ± standard deviation (m±SD), while categorical data was expressed as percentages (%). For continuous data between two groups following a normal distribution, an independent samples t-test was utilized for comparison, and within-group comparisons were conducted using a paired samples t-test. We employed repeated measures analysis of variance to analyze clinical outcomes at different time points. In cases where normal distribution assumptions were not met, the Mann-Whitney U test was employed. The categorical data between groups was compared using the chi-square test. We used the intraclass correlation coefficient (ICC) to evaluate the inter-observer variability. Statistical significance was considered when p ≤ 0.05.

### Part II finite element model (FEM) analysis

#### Development of healthy lumbar spine model

**FIGURE 1:**
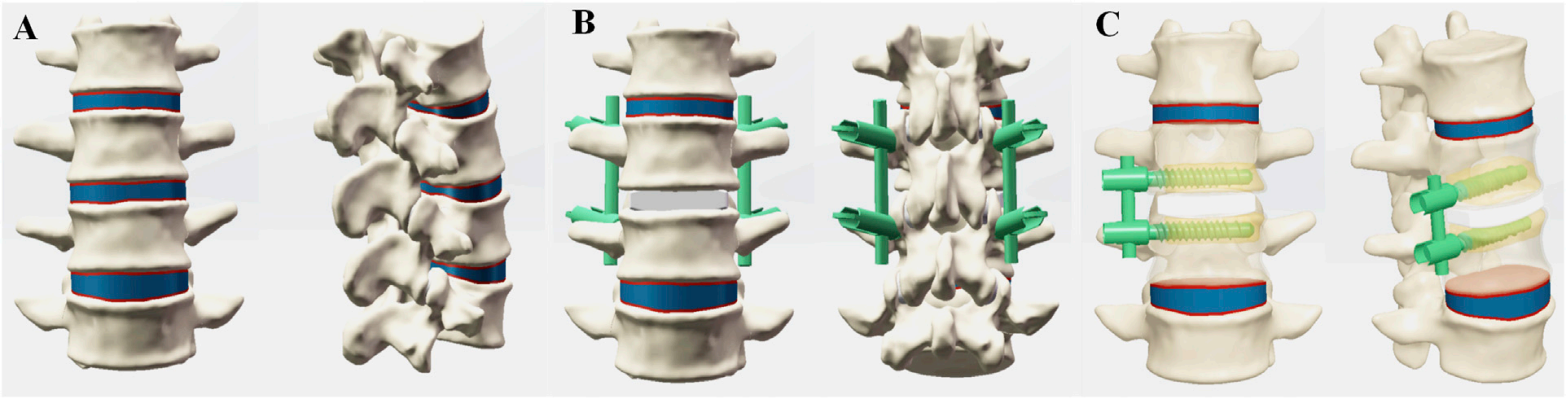
Finite element (FE) models in the current study. **(A)** FE model of the intact L2-L5 spine, **(B)** FE model of the OLIF-PIFPS, **(C)** FE model of the OLIF-BCAAS.

We selected the CT scans of a healthy male subject (age: 31 years old, height: 175 cm, weight: 76 kg) with no spinal diseases to construct a three-dimensional nonlinear FE model of the L2-5 segment. The data from lumbar thin-layer CT (a slice thickness of 1 mm) was imported into Mimics software (Version 21, Materialise Inc., Leuven, Belgium), and pixels within the grayscale values range of 226–1,300 were selected. Subsequently, the vertebrae bodies of L2, L3, L4, and L5 were segmented following appropriate orientations and sequences. The preliminary 3-dimensional (3D) models underwent wrapping and smoothing processes, after which the L2-5 vertebrae were exported as STL files. The sketchy model was imported into Geomagic Studio software (Version 12, Geomagic Inc., Cary, North Carolina, United States) for surface repair of the vertebral models and further processing, then producing a more elaborate 3D solid model. According to the anatomical characteristics of the lumbar spine, the components including the cortical bone, trabecular bone, endplate, nucleus pulposus, annulus fibrosus, and articular cartilage were assembled in the Parts Interface window of the SolidWorks 2020 software (Dassault Systemes Americas Corp., Waltham, Massachusetts, United States). In ANSYS ICEM CFD (Version 19.0, Ansys, Ltd., Canonsburg, Pennsylvania, United States), we utilized spring elements as substitutes for various ligament structures in the model to replicate the ligaments between the vertebras (Figure 1A).

#### Development of the OLIF surgical models

The model consists of 4 vertebral bodies and three intervertebral discs. To construct the OLIF surgical model, we removed part of the annulus and the entire nucleus pulposus while preserving the superior and inferior endplates. Then, an established OLIF cage with 50 × 12 × 18 mm (length × height × width) was implanted into the defective disc from the left side of the vertebral body. The anterolateral screws had a length of 50 mm and a diameter of 6.0 mm, while a connection rod had a diameter of 5.5 mm and a length of 50 mm (Figure 1B). The pedicle screws were a diameter of 4.5 mm and a length of 60 mm, with the rods of 5.5 mm in diameter and 50 mm in length, respectively. The implantation of bone cement and internal fixation systems adhered to the established standard technical procedures (Zhong et al., 2023; Ge et al., 2023) (Figure 1C). The osteoporotic model in this study was established by adjusting material properties. Osteoporotic material properties were defined as a 66% reduction in elastic modulus for cancellous bone and a 33% reduction for cortical bone, endplates, and posterior structures, while maintaining the soft tissue structures unchanged (Polikeit et al., 2003).

#### Material properties, boundary conditions, and loading

In this study, the mechanical properties of all lumbar elements were derived from the previous studies (Kim et al., 2014; Lu and Lu, 2019), as detailed in Table 1. The inferior endplate of L5 was fixed to prevent any displacement or rotation under load. A vertical load of 500 N and a moment load of 7.5 N·m were applied to the superior surface of L3 to simulate physiological spinal activities, including flexion, extension, left and right lateral bending, and left and right rotation. The FE models were analyzed under different loading conditions to evaluate: 1) segmental L3/4 range of motion (ROM); 2) stress on L3 inferior and L4 superior endplates; 3) cage and supplemental fixation stress; and 4) L3 maximum displacement with respect to L4.

**TABLE 1:** Material properties of the finite element models.

Components	Young’s modulus (Mpa)	Poisson’s ratio
Cortical bone	12,000	0.3
Cancellous bone	100	0.3
Posterior elements	3,500	0.25
Cortical endplate	12,000	0.3
Annulus ground	Mooney-Rivlin, C1 = 0.18, C2 = 0.045	-
Nucleus pulposus	Mooney-Rivlin, C1 = 0.12, C2 = 0.03	-
Annulus fibrosus	Calibrated stress-strain curves	-
Ligaments	Calibrated defection-force curves	0.3
Cage (PEEK)	3,600	0.25
Bone cement	3,000	0.41
Pedicle screw and rods	110,000	0.28
Anterolateral screws	110,000	0.28

#### Model validation

We applied a 500 N axial compression preload and a moment load of 7.5 N·m to the intact lumbar spine model. Subsequently, we measured the range of motion (ROM) of the L3/4 segment in six directions: flexion, extension, left and right lateral bending, and left and right axial rotation. The results for the intact model were compared with the data from the cadaveric experimental study conducted by Fiebert et al. (1989). Moreover, our data for the intact model remain in alignment with previously published studies (Zhong et al., 2023; Sin and Heo, 2019) under identical experimental settings. Therefore, the FE models of the present study were validated, allowing its further use in biomechanical analysis of the lumbar spine.

## Results

### Clinical study

Seventeen females and six males were enrolled in the OLIF-BCAAS group, with a mean age of (73.65 ± 5.24) years (ranging from 63 to 83), a mean BMI of (24.07 ± 2.92) (ranging from 18.5 to 29.2) and a mean BMD of (−3.33 ± 0.47) (ranging from −2.6 to −4.3). Within this cohort, 14 cases underwent lumbar surgery at the level of L4/5, 7 cases at the level of L3/4, and 2 cases at the level of L2/3. There were seventeen females and eight males in the OLIF-PIFPS group, with a mean age of (72.40 ± 4.91) years (ranging from 65 to 84), a mean BMI of (23.61 ± 2.63) (ranging from 19.1 to 29.4) and a mean BMD of (−3.42 ± 0.39) (ranging from −2.6 to −4.2). Within this cohort, 13 cases underwent lumbar surgery at the level of L4/5, 9 cases at the level of L3/4, and 3 cases at the level of L2/3. Demographic data, including patients’ age, gender, BMI, BMD, and surgical spine level, were also similar between the two groups (Table 2).

**TABLE 2:** Demographic background and surgical related parameters.

	OLIF-BCAAS (n = 23)	OLIF-PIFPS (n = 25)	*p* value
Age (years)	73.65 ± 5.24	72.40 ± 4.91	0.397
Gender			
Male	6	8	0.653
Female	17	17	
BMI (kg/m^2^)	24.07 ± 2.92	23.61 ± 2.63	0.567
BMD (T-value)	−3.33 ± 0.47	−3.42 ± 0.39	0.455
Spinal level			
L2/3	2	3	0.817
L3/4	7	9	
L4/5	14	13	
OP time (min)	84.35 ± 13.25	121.20 ± 16.35	0.000
Blood loss (mL)	54.13 ± 20.04	90.80 ± 16.44	0.000
Hospitalization (d)	4.17 ± 1.07	4.64 ± 0.76	0.087

OLIF-BCAAS, oblique lateral interbody fusion with bone cement-augmented anterolateral screws; OLIF-PIFPS, oblique lateral interbody fusion combined with posterior internal fixation with pedicle screws; BMI, body mass index; BMD, bone mineral density; OP, operative. Data presented as mean ± standard deviation.

The mean operative time and intraoperative blood loss in the OLIF-BCAAS group were (84.35 ± 13.25) minutes and (54.13 ± 20.04) mL, respectively, compared to (121.20 ± 16.35) minutes and (90.80 ± 16.44) in the OLIF-PIFPS group, showing significant differences between the two groups (p < 0.01). However, there was no significant difference in hospitalization time among the two groups [OLIF-BCAAS group: (4.17 ± 1.07) days, OLIF-PIFPS group: (4.64 ± 0.76) days, p = 0.087, Table 2].

In the OLIF-BCAAS group, the mean VAS-lower back and VAS-leg scores decreased significantly from preoperative levels of (6.74 ± 0.96) and (6.09 ± 0.85) to (2.43 ± 0.73) and (2.70 ± 0.93) at 3 months postoperatively (p < 0.05), with a slight further decrease observed at 6 and 12 months postoperatively. The comparable trends in the VAS-lower back and VAS-leg scores were also noted in the OLIF-PIFPS group. Significant differences in the VAS-lower back score were found at 3 months and 12 months postoperatively (p < 0.05, Table 3), with a trend towards statistical difference at 6 months postoperatively among the two groups (p = 0.054, Table 3).

**TABLE 3:** Clinical evaluation of the patients in two groups.

	OLIF-BCAAS (n = 23)	OLIF-PIFPS (n = 25)	*p* value
VAS-lower back score			
Pre-	6.74 ± 0.96	6.64 ± 1.08	0.739
Post- 3 months	2.43 ± 0.73	3.12 ± 0.88	0.005
Post- 6 months	1.48 ± 0.73	1.92 ± 0.81	0.054
Post- 12 months	1.30 ± 0.56	1.84 ± 0.80	0.011
*F* value	494.197	433.414	
*p* value	0.000	0.000	
VAS-leg score			
Pre-	6.09 ± 0.85	6.28 ± 0.98	0.471
Post- 3 months	2.70 ± 0.93	2.64 ± 0.86	0.830
Post- 6 months	1.43 ± 0.66	1.48 ± 0.71	0.822
Post- 12 months	1.22 ± 0.42	1.28 ± 0.61	0.685
*F* value	275.635	376.311	
*p* value	0.000	0.000	
JOA score			
Pre-	13.52 ± 2.00	13.64 ± 1.87	0.833
Post- 3 months	20.13 ± 1.89	19.96 ± 1.99	0.763
Post- 6 months	22.78 ± 1.88	22.56 ± 2.08	0.700
Post- 12 months	24.39 ± 2.17	24.16 ± 1.86	0.693
*F* value	545.013	594.496	
*p* value	0.000	0.000	
IR (%)	71.35 ± 10.99	69.28 ± 9.23	0.483

OLIF-BCAAS, oblique lateral interbody fusion with bone cement-augmented anterolateral screws; OLIF-PIFPS, oblique lateral interbody fusion combined with posterior internal fixation with pedicle screws; VAS, visual analogue scale; Pre-, preoperative; Post-, postoperative; mons, months; JOA, japanese orthopaedic association; IR, improvement rate. Data presented as mean ± standard deviation.

The mean JOA score of the OLIF-BCAAS group improved significantly from (13.52 ± 2.00) preoperatively to (20.13 ± 1.89) at 3 months postoperatively, and continued to improve gradually to (22.78 ± 1.88) at 6 months postoperatively and (24.39 ± 2.17) at 12 months postoperatively. A similar trend was also observed in the OLIF-PIFPS group. There were no significant differences in JOA scores preoperatively, postoperatively, or improvement rates between the two groups (p > 0.05, Table 3).

Two authors (X.P.M. and X.D.W.) independently measured/evaluated the radiological images. Inter-observer variability was counted among them for DH, CS and fusion status. The excellent agreement was observed for CS (ICC value: 0.86) and CS (ICC value: 0.81). The substantial agreement between two authors on DH (ICC value: 0.73).

The preoperative mean DH in group OLIF-BCAAS was (8.40 ± 0.90) mm, (8.38 ± 1.01) mm in group OLIF-PIFPS, with no significant difference between the two groups (p > 0.05). DH in both groups significantly increased 1 day postoperatively, followed by varying degrees of reduction at 6 and 12 months postoperatively. However, the decrease in DH at 12 months postoperatively was more significant in the OLIF-PIFPS group compared to the OLIF-BCAAS group (p < 0.05, Table 4). No significant difference in the change of DH from postoperative 1 day to preoperatively (△DH Pre- Post 1 day) was detected among the groups. However, △DH Post 1 day-12 m of the OLIF-BCAAS group was statistically lower than that of the OLIF-PIFPS group (p < 0.05, Table 4).

**TABLE 4:** Radiographic evaluation of the patients in two groups.

	OLIF-BCAAS (n = 23)	OLIF-PIFPS (n = 25)	*p* value
DH (mm)			
Pre-	8.40 ± 0.90	8.38 ± 1.01	0.930
Post- 1 day	10.90 ± 1.03	11.00 ± 1.31	0.790
Post- 6 months	10.03 ± 0.84	9.88 ± 1.00	0.599
Post- 12 months	9.38 ± 0.78	8.86 ± 0.91	0.042
*F* value	86.170	108.668	
*p* value	0.000	0.000	
△DH _Pre- Post 1d_	2.50 ± 0.99	2.62 ± 0.81	0.657
△DH _Post 1d–12m_	1.53 ± 0.81	2.13 ± 1.05	0.031
CS _Post 12m-1d_			
None	18 (78.26%)	13 (52.00%)	0.057
Mild	4 (17.39%)	9 (36.00%)	
Severe	1 (4.35%)	3 (12.00%)	
Fusion			
Post- 6 months	10 (43.48%)	12 (48.00%)	0.780
Post- 12 months	18 (78.26%)	19 (76.00%)	0.852

OLIF-BCAAS, oblique lateral interbody fusion with bone cement-augmented anterolateral screws; OLIF-PIFPS, oblique lateral interbody fusion combined with posterior internal fixation with pedicle screws; DH, disc height; Pre-, preoperative; Post-, postoperative; d, day; mons, months; △DH, _Pre- Post 1d_, change of DH, from postoperative 1 day to preoperatively; △DH, _Post 1d–12m_, change of DH, from postoperative 12 months to 1 day postoperatively; CS, cage subsidence. Data presented as mean ± standard deviation.

**FIGURE 2:**
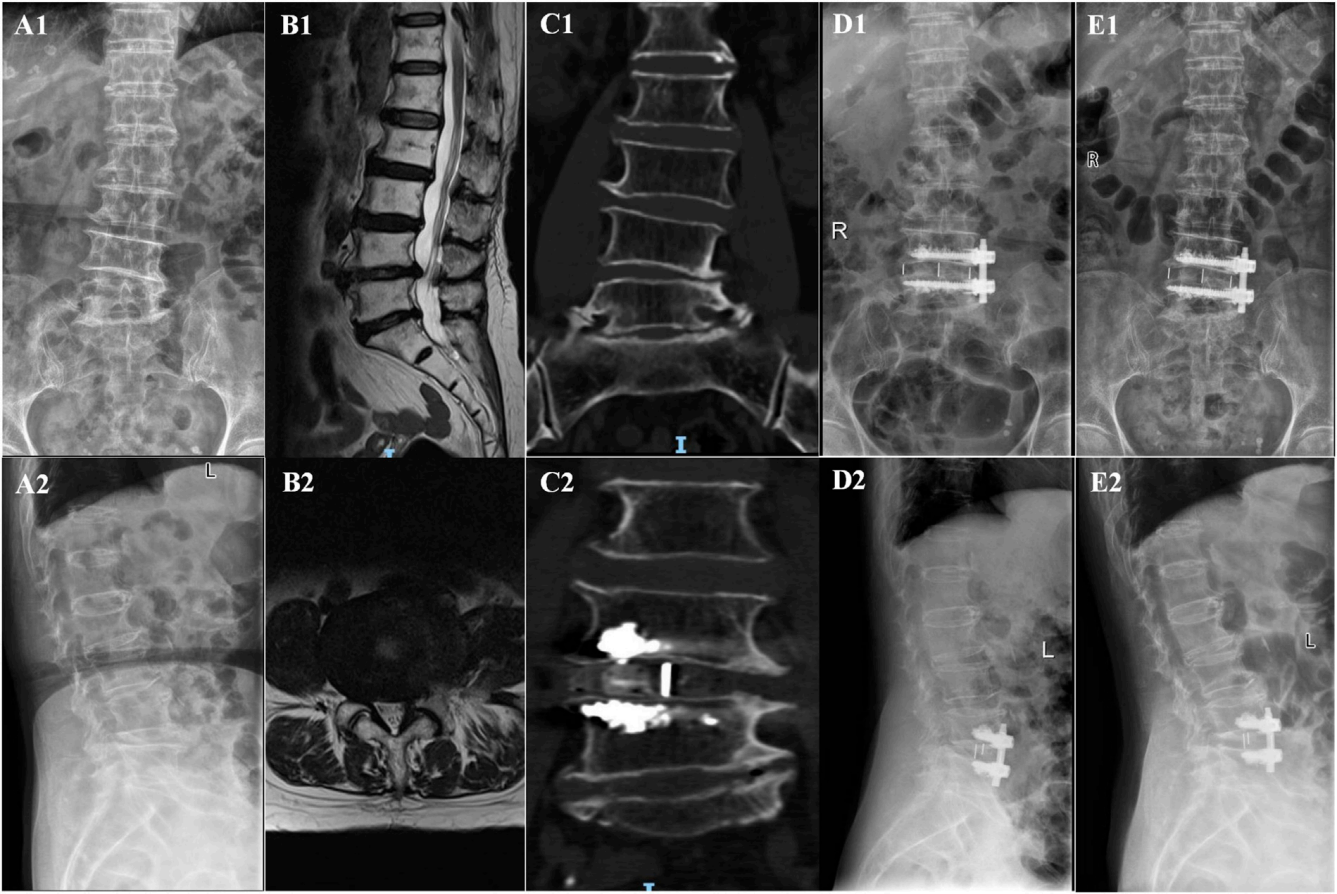
The radiological images of OLIF-BCAAS typical case. **(A1,A2)** Preoperative A−P, lateral X-ray film; **(B1,B2)** Preoperative sagittal and axial MR images; **(C1,C2)** Preoperative and postoperative coronal CT; **(D1,D2)** A−P and lateral X-ray film at 1 day postoperatively; **(E1,E2)** A−P, lateral X-ray film at last follow-up postoperatively.

**FIGURE 3:**
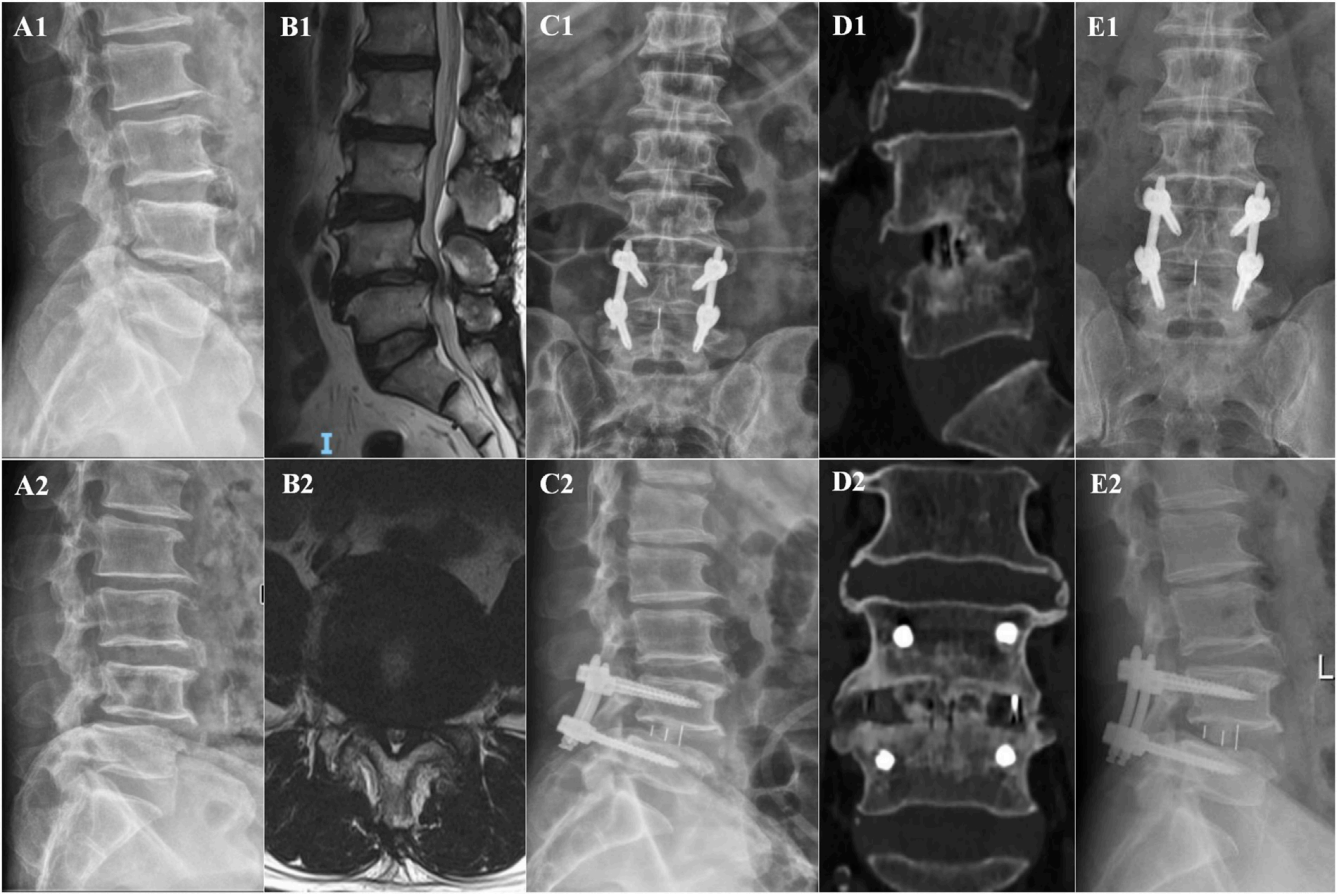
The radiological images of OLIF-PIFPS typical case. **(A1,A2)** Preoperative dynamic X-ray films; **(B1,B2)** Preoperative sagittal and axial MR images; **(C1,C2)** A−P, lateral X-ray film at 1 day postoperatively; **(D1,D2)** Sagittal and coronal CT images at 12 months postoperatively; **(E1,E2)** A−P, lateral X-ray film at 12 months postoperatively.

In the OLIF-BCAAS group, five cases (5/23, 21.74%) of CS were detected, with 4 out of 23 cases (17.39%) considered as mild CS. In contrast, the amount of CS was 12 cases (12/25, 48%) in the OLIF-PIFPS group, which included 3 cases of severe CS. There was a trend towards statistical difference in CS between the two groups (p = 0.057, Table 4). However, no significant differences were found in fusion rate at 6 months and 12 months postoperatively among the groups (p > 0.05, Table 4). Figures 2, 3 show the radiological images of the OLIF-BCAAS and OLIF-PIFPS typical cases.

In this work, we conducted a statistical power analysis to assess inter-group and intra-group differences in VAS scores using G*Power software (version 3.1, Heinrich-Heine-Universität Düsseldorf, Germany). This was done to confirm that the sample size was sufficient to detect the target effect. The statistical analysis revealed that the power for detecting differences in VAS scores at 12 months postoperative was 85%, suggesting that the current sample size is adequate to detect a medium-to-large effect (Cohen’s d = 0.776). Based on conclusions from previous studies, we set the correlation coefficient (r) at 0.60 (Nakajima et al., 2025) and considered a reduction of 2.0 points in the VAS score as the Minimal Clinically Important Difference (MCID) (Fishbain et al., 2015). The intra-group VAS scores at 12 months postoperative showed a significant decrease compared to preoperative values, with an extremely large effect size (Cohen’s d > 5) and statistical power approaching 100%. These findings indicate that the observed pain relief after treatment has both strong statistical and clinical significance.

### FEM analysis

#### Segmental L3/4 ROM

**FIGURE 4:**
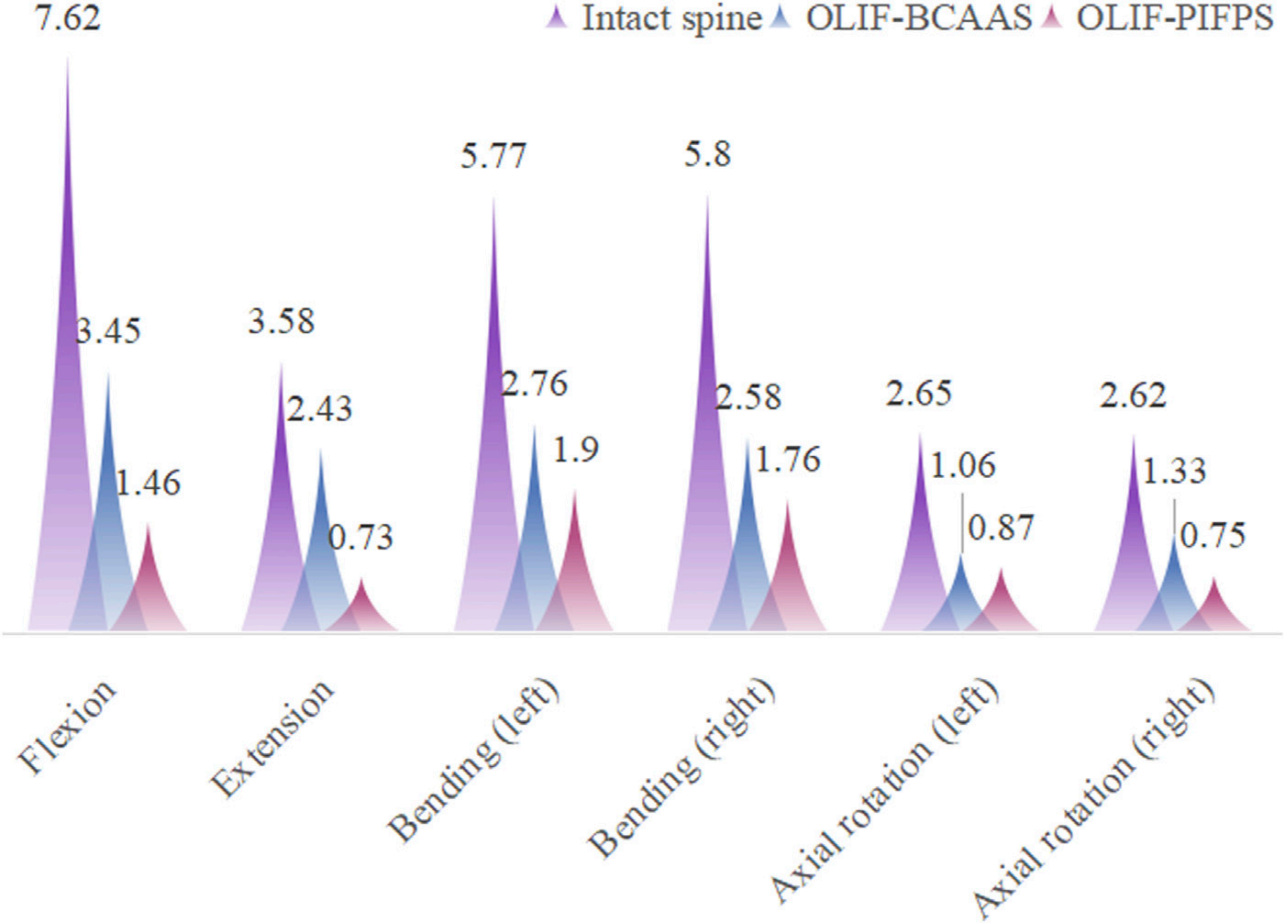
The ROM of the L3/4 segment in the intact lumbar model and both OLIF surgical models under six motion modes.

The surgical segment for undergoing lumbar fusion and internal fixation was determined to be L3/4 in the current study; hence, we only investigated the ROM of L3/4 for the intact lumbar and both OLIF surgical models. The ROM of the L3/4 segment in the intact lumbar model and both OLIF surgical models under six motion modes are shown in Figure 4. Compared to the intact lumbar model, both OLIF surgical models significantly reduced the ROM of L3/4. Relative to the intact lumbar model, the L3/4 ROM of the OLIF-BCAAS model decreased by 54.72%, 32.12%, 52.17%, 55.52%, 60.00%, and 49.24% in flexion, extension, left bending, right bending, left axial rotation, and right axial rotation, respectively. Similarly, the OLIF-PIFPS model exhibited reductions of 80.84%, 79.61%, 67.07%, 69.66%, 67.17%, and 71.37% in the six motions mentioned above modes. Furthermore, compared to the OLIF-BCAAS model, the reduction of the L3/4 ROM in the OLIF-PIFPS model was more pronounced in flexion, extension, left and right bending, and left and right axial rotation, indicating that lumbar levels underwent OLIF-PIFPS have less mobility.

#### Stress on L3 inferior and L4 superior endplates

**FIGURE 5:**
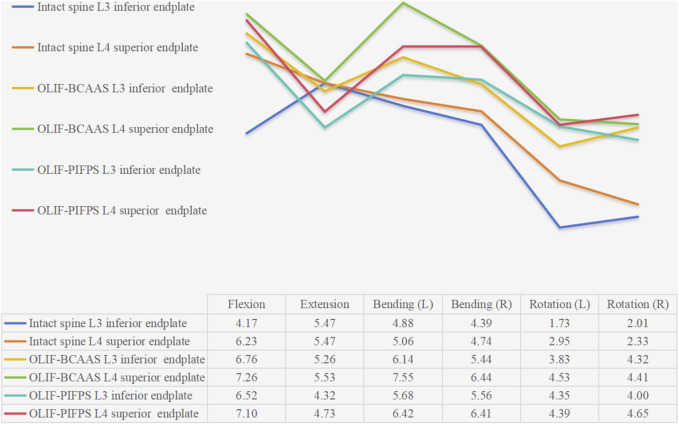
Line graph of the stress distribution on the L3 inferior and L4 superior endplates for the intact lumbar model and two OLIF surgical models under six motion modes.

**FIGURE 6:**
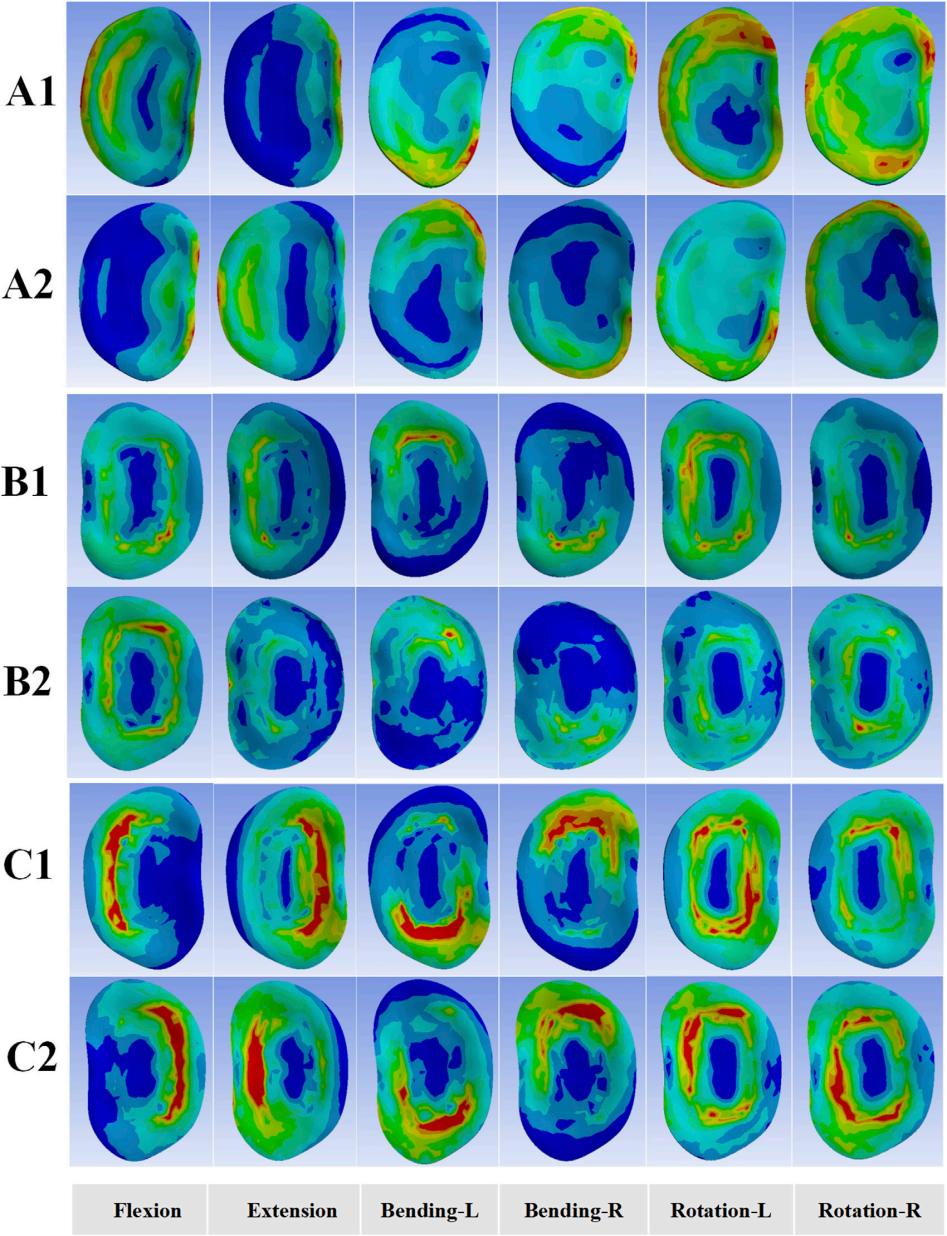
Endplates stress distribution for the intact lumbar FE model and two OLIF surgical FE models under six motion modes. **(A1,A2)** Stress distributions of the L3 inferior and L4 superior endplates for the intact lumbar model; **(B1,B2)** Stress distributions of the L3 inferior and L4 superior endplates for the OLIF-BCAAS model; **(C1,C2)** Stress distributions of the L3 inferior and L4 superior endplates for the OLIF-PIFPS model.

Figure 5 shows the stress distribution on the L3 inferior and L4 superior endplates for the intact lumbar model and two OLIF surgical models under six motion modes. In all motion modes, the stress on the L4 superior endplate was higher than that on the L3 interior endplate, predominantly concentrating at the edges of the endplates (Figure 6). Moreover, except for extension motion, both OLIF surgical models exhibited significantly higher stress on the L3 inferior and L4 superior endplates than the intact lumbar model in the other five motion modes. However, no significant differences in the stress on the L3 inferior and L4 superior endplates were documented in the two OLIF surgical models under six motion modes.

#### Cage and supplemental fixation stress

**FIGURE 7:**
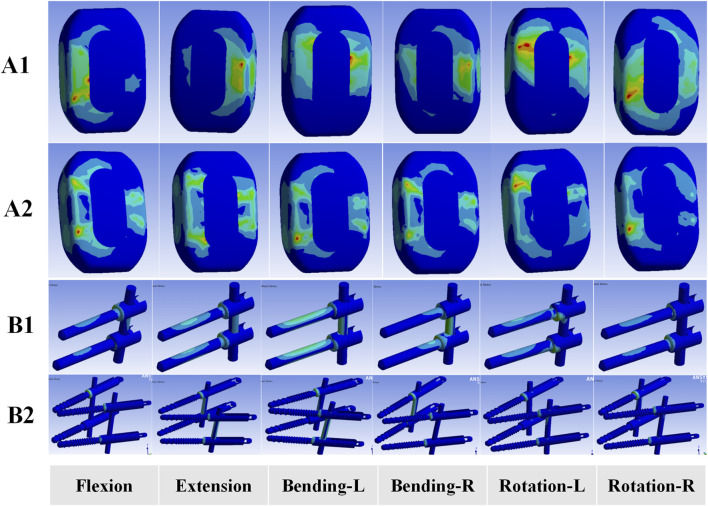
Stress distributions of cage and supplemental fixation system for the two OLIF surgical models. **(A1)** Stress distributions of cage for OLIF-BCAAS; **(A2)** Stress distributions of cage for OLIF-PIFPS; **(B1)** Stress distributions of supplemental fixation system for OLIF-BCAAS; **(B2)** Stress distributions of supplemental fixation system for OLIF-PIFPS. L, left; R, right.

**FIGURE 8:**
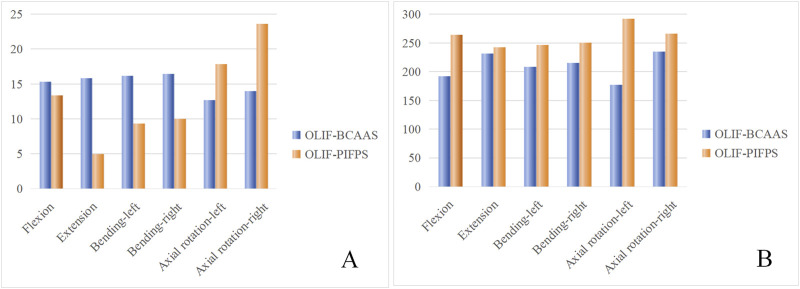
Hostogram of the stress on the cage **(A)** and supplementary fixation systems **(B)** of the two OLIF surgical models.

Figure 7 illustrates the stress on the cage and supplemental fixation systems of the two OLIF surgical models. The stress on the cage in both OLIF surgical models was predominantly concentrated at the edges of the cage under six motion modes. During flexion, extension, and lateral bending, the cage stress was notably higher in the OLIF-BCAAS model compared to the OLIF-PIFPS model. However, the cage stress in the OLIF-PIFPS model surpassed that in the OLIF-BCAAS model under rotational motion (Figure 8A). Compared to the OLIF-PIFPS model, the supplemental fixation system of the OLIF-BCAAS model exhibited lower stress under all six motion modes (Figure 8B).

#### L3 maximum displacement with respect to L4

**FIGURE 9:**
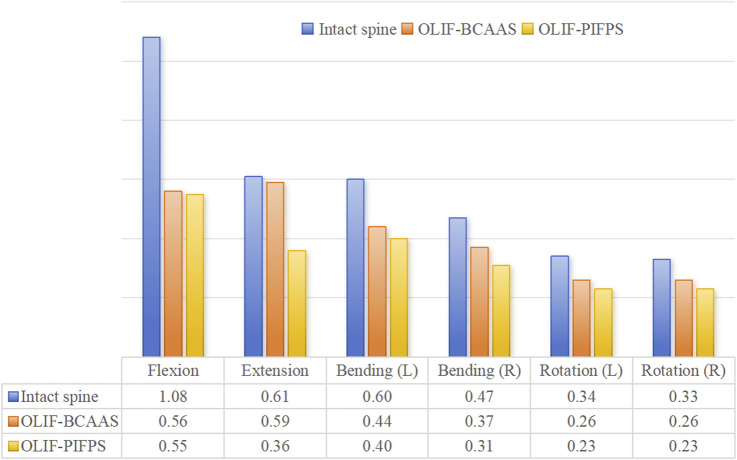
The L3 maximum displacement with respect to L4 for the two OLIF surgical models under six motion modes.

The maximum intervertebral displacement of the superior vertebra body relative to the inferior vertebra body indirectly evaluated the stability of the internal fixation system and the surgical segment. Although the reliability of the relative displacement was not as robust as that of ROM, it still served as an adjunct criterion for evaluating lumbar stability (Eskandari et al., 2019). Theoretically, smaller relative displacements indicated more excellent stability of the internal fixation system. Compared to the intact lumbar model, both OLIF surgical models demonstrated significantly reduced L3 maximum displacement under the six motion models. However, the reduction was more pronounced in the OLIF-PIFPS model than in the OLIF-BCAAS model (Figure 9).

## Discussion

As the population ages, there is an increasing trend in patients with LDD complicated by osteoporosis seeking lumbar fusion surgery. However, standardized strategies among the minimally invasive surgical procedures for such populations still need to be improved in clinical practice. Currently, few studies are exploring the innovative combination of OLIF with bone cement augmentation technique in the clinical management of LDD associated with osteoporosis. To our knowledge, this is the first study that integrates clinical and biomechanical approaches to investigate and demonstrate the effectiveness and safety of OLIF-BCAAS in patients with LDD complicated by osteoporosis.

The main findings of the current study are as follows: 1) OLIF-BCAAS exhibited shorter surgical times, reduced blood loss, and lower levels of back pain within 1 year postoperatively compared to OLIF-PIFPS. 2) Both OLIF surgical techniques showed significant improvements in DH postoperatively; however, the reduction in DH at 12 months after surgery was more pronounced in the OLIF-PIFPS group than in the OLIF-BCAAS group. 3) Both procedures achieved good fusion postoperatively, although the incidence of CS appeared to be lower or slight in the OLIF-BCAAS group. 4) The results of FE model analysis demonstrated that both OLIF surgical models exhibited significant reductions in local L3/4 ROM and L3 maximum displacement with respect to L4 under six motion patterns when compared to a complete lumbar model, with the OLIF-PIFPS model showing a more pronounced decline. 5) In the OLIF-BCAAS model, the stress on the endplates and cages was primarily concentrated in the surrounding regions and was higher than that in the OLIF-PIFPS model; however, the stress on the supplemental fixation devices was significantly lower than that observed in the OLIF-PIFPS model.

OLIF is a minimally invasive surgical technique that entails the resection of the intervertebral disc through an oblique anterior incision, followed by the insertion of a large cage to restore intervertebral height, thereby facilitating indirect decompression (Hung et al., 2021). This procedure is further enhanced by anterolateral or posterior screw fixation to reconstruct lumbar stability. In the general population, the postoperative incidence of CS varies between 8.8% and 46.7% (Kotheeranurak et al., 2023). Previous clinical study (Ma et al., 2023) and biomechanical research (Bereczki et al., 2021) have demonstrated that the combination of OLIF with posterior pedicle screw fixation can effectively mitigate the occurrence of CS. However, the intraoperative changes between the lateral and prone positions undoubtedly prolong surgical and anesthesia times (Li Z. et al., 2023). Furthermore, the damage to the posterior structures of the lumbar spine caused by the implantation of the posterior screw-rod system may account for the significantly greater improvement in back pain observed in the OLIF-BCAAS group compared to the OLIF-PIFPS group within 1 year postoperatively in this study.

The integrity of the endplates and their capacity to withstand mechanical stress are critical determinants of postoperative reductions in DH and the incidence of CS (Park et al., 2019). Furthermore, supplemental internal fixation can prevent CS by improving segment stability and alleviating stress on the cage (Fan et al., 2023). The FE analysis conducted in the present study demonstrated that both OLIF surgical models exhibited commendable internal fixation performance; however, the OLIF-PIFPS model showed superior fixation strength. Theoretically, when more stress is applied to the supplemental internal fixation device, the stress experienced by the endplates and cages of the lumbar segment decreases accordingly. This observation may account for the higher stresses recorded in the endplates and cages of the OLIF-BCAAS model group compared to the OLIF-PIFPS model, despite significantly lower stress levels on the supplemental fixation device in the OLIF-PIFPS model. Consequently, one might reasonably assume that the incidence of postoperative CS would be greater in the OLIF-BCAAS group. Contrarily, the findings from our clinical part indicated that patients undergoing the OLIF-BCAAS experienced a lower incidence of CS. We speculate that: 1) OLIF-BCAAS transforms the traditional stress transfer pattern between the vertebral body and screw at two interfaces into a three-interface system involving the vertebral body, screw, and bone cement, thus more effectively enhancing local stability in the lumbar region; and 2) the reinforced endplates with bone cement possess better stress-bearing capacity, effectively counteracting the cutting effects of the cage on the endplates. Furthermore, the application of a modest amount of well-dispersed bone cement does not negatively impact the fusion rate associated with this procedure (Peng et al., 2024).

Osteoporosis is recognized as a significant contributor to the decline in vertebral strength and the diminished capacity of the endplates to endure mechanical stress, both of which are established as critical risk factors for CS following lumbar fusion surgery (Kotheeranurak et al., 2023; Pu et al., 2023). While a certain degree of DH reduction may facilitate fusion by increasing the contact area between the cage and the endplates, the cage invasion into the vertebral body caused by CS frequently leads to persistent low back pain (Hou and Yuan, 2012). The development of OLIF-BCAAS signifies an innovative integration of the OLIF technique with bone cement augmentation. The present work indicated that OLIF-BCAAS not only inherited the minimally invasive natures of the OLIF technique (Emami et al., 2023), but also leveraged the advantages of bone cement augmentation in reducing the incidence of postoperative CS in patients with osteoporosis (Deml et al., 2022). Recently, two retrospective studies by Zeng et al. (Li Z. et al., 2023; Peng et al., 2024) sought to explore the safety and efficacy of OLIF combined with stress endplate augmentation and anterolateral screw fixation for degenerative lumbar spinal stenosis with osteoporosis, revealing clinical and radiological findings consistent with those of the present study. However, it is essential to note that patients in their control group underwent OLIF in conjunction with anterolateral screw fixation, which is not advisable for patients with osteoporosis due to its association with elevated rates of CS and symptom recurrence postoperatively.

Currently, there is no consensus on the choice of internal fixation for patients with osteoporosis. The selection of various internal fixations largely depends on the clinical experience of the surgeons and their preferences for specific techniques. The use of cement-augmented screws has been shown to significantly improve the stability of the screws (Mo et al., 2019). A recent study (Kim J. H. et al., 2020) compared the clinical application of hollow and traditional cemented pedicle screws in osteoporotic populations and reported a lower incidence of postoperative screw loosening. Moreover, previous study (Liu et al., 2013) have reported that expensive pedicle screws provide better stability than cemented pedicle screws. Although these adjunctive fixation devices can make screws more stable and secure, complications and the associated technical complexity limit their widespread clinical application.

This study has certain limitations. Firstly, the nature of the retrospective study inherently resulted in a lower evidence level for the conclusions drawn compared to prospective randomized controlled trials (RCTs). However, it is worth noting that our institutional ethics committee has approved the study protocol of an RCT on this topic and is currently in the participant recruitment phase. Secondly, the clinical part of the present work was constrained by a relatively small sample size and a limited follow-up period. Furthermore, ODI score is the importance in evaluating the effectiveness of lumbar surgery, particularly its irreplaceable value in the comprehensive assessment of patients’ functional status and quality of life. VAS score objectively quantifies the degree of pain relief and is a core dimension in assessing surgical outcomes. The JOA score, which evaluates improvements in motor, sensory, and bladder function, partially overlaps with the ODI score in functional assessment. However, this study was a retrospective analysis, and data collection was limited by the content of the original medical records. Although the present study incorporated internationally recognized VAS and JOA scores as core metrics, ODI score was not systematically recorded in clinical practice, which has led us to exercise caution in interpreting the clinical outcomes of this work. Moreover, our FE model analysis did not reconstruct the paravertebral soft tissue to assess the impact of muscles on spinal biomechanical function, which is a common issue in such analyses. Nonetheless, we employed a more rigorous methodology than previous studies to validate the FE model and to simulate various scenarios under consistent experimental conditions. Therefore, our model effectively evaluated the biomechanical performance of the two OLIF surgical techniques.

## Conclusion

The current study demonstrates that both OLIF surgical techniques for treating LDD combined with osteoporosis can achieve favorable clinical outcomes. However, OLIF-BCAAS exhibits more significant advantages over OLIF-PIFPS by maximizing the benefits of minimally invasive surgery, which include reduced trauma, decreased bleeding, and shorter operation times. Moreover, OLIF-BCAAS is associated with lower levels of postoperative back pain and a reduced incidence of postoperative CS.

## Data Availability

The raw data supporting the conclusions of this article will be made available by the authors, without undue reservation.

## References

[B1] Bereczki F. Turbucz M. Kiss R. Eltes P. E. Lazary A. (2021). Stability evaluation of different oblique lumbar interbody fusion constructs in normal and osteoporotic condition-a finite element based study. Front. Bioeng. Biotechnol. 9, 749914. 10.3389/fbioe.2021.749914 34805108 PMC8602101

[B2] Bridwell K. H. Lenke L. G. McEnery K. W. Baldus C. Blanke K. (1995). Anterior fresh frozen structural allografts in the thoracic and lumbar spine.Do they work if combined with posterior fusion and instrumentation in adult patients with kyphosis or anterior column defects? Spine. Spine (Phila. pa. 1976.) 20, 1410–1418. 10.1097/00007632-199506020-00014 7676341

[B3] Cai K. Luo K. Zhu J. Zhang K. Yu S. Ye Y. (2021). Effect of pedicle-screw rod fixation on oblique lumbar interbody fusion in patients with osteoporosis: a retrospective cohort study. J. Orthop. Surg. Res. 16, 429. 10.1186/s13018-021-02570-8 34217340 PMC8254285

[B4] Canyang H. Weidong C. Zhiping H. Xiuhua W. Minghui Z. Haihong H. (2023). A novel semi-cannulated screw enhanced bone cement augmentation and pullout strength in posterior cervical lateral mass screw fixations: an *in vitro* biomechanical and morphological study. Orthop. Surg. 15, 2927–2936. 10.1111/os.13859 37712328 PMC10622296

[B5] Deml M. C. Cattaneo E. N. Bigdon S. F. Sebald H. J. Hoppe S. Heini P. (2022). PMMA-Cement-PLIF is safe and effective as a single-stage posterior procedure in treating pyogenic erosive lumbar spondylodiscitis-a single-center retrospective study of 73 cases. Bioeng. (Basel) 9, 73. 10.3390/bioengineering9020073 PMC8869766 35200426

[B6] Elder B. D. Lo S. F. Holmes C. Goodwin C. R. Kosztowski T. A. Lina I. A. (2015). The biomechanics of pedicle screw augmentation with cement. Spine J. 15, 1432–1445. 10.1016/j.spinee.2015.03.016 25797809

[B7] Elmose S. F. Andersen G. O. Carreon L. Y. Sigmundsson F. G. Andersen M. O. (2023). Radiological definitions of sagittal plane segmental instability in the degenerative lumbar spine - a systematic review. Glob. Spine J. 13, 523–533. 10.1177/21925682221099854 PMC9972266 35606897

[B8] Emami A. Patel N. Coban D. Saela S. Sinha K. Faloon M. (2023). Comparing clinical and radiological outcomes between single-level OLIF and XLIF: a systematic review and meta-analysis. N. Am. Spine Soc. J. 14, 100216. 10.1016/j.xnsj.2023.100216 37234475 PMC10205548

[B9] Eskandari A. H. Arjmand N. Shirazi-Adl A. Farahmand F. (2019). Hypersensitivity of trunk biomechanical model predictions to errors in image-based kinematics when using fully displacement-control techniques. J. Biomech. 84, 161–171. 10.1016/j.jbiomech.2018.12.043 30638978

[B10] Fan K. Zhang D. Xue R. Chen W. Hou Z. Zhang Y. (2023). Biomechanical analysis of double-level oblique lumbar fusion with different types of fixation: a finite element-based study. Orthop. Surg. 15, 1357–1365. 10.1111/os.13703 37073100 PMC10157704

[B11] Fiebert I. M. Spyropoulos T. Peterman D. Oxland T. (1989). Three-dimensional movements of the whole lumbar spine and lumbosacral joint. Spine (Phila Pa 1976) 14, 1256–1260. 10.1097/00007632-198911000-00020 2603060

[B12] Fishbain D. A. Gao J. Lewis J. E. Zhang L. (2015). At completion of a multidisciplinary treatment program, are psychophysical variables associated with a VAS improvement of 30% or more, a minimal clinically important difference, or an absolute VAS score improvement of 1.5 cm or more? Pain Med. 17, 781–789. 10.1093/pm/pnv006 26814242

[B13] Formby P. M. Kang D. G. Helgeson M. D. Wagner S. C. (2016). Clinical and radiographic outcomes of transforaminal lumbar interbody fusion in patients with osteoporosis. Glob. Spine J. 6, 660–664. 10.1055/s-0036-1578804 PMC5077707 27781185

[B14] Ge T. Hu B. Zhang Q. Xiao J. Wu X. Xia D. (2023). Biomechanical evaluation of two-level oblique lumbar interbody fusion combined with posterior four-screw fixation:A finite element analysis. Clin. Neurol. Neurosurg. 225, 107597. 10.1016/j.clineuro.2023.107597 36696847

[B15] Hines C. B. (2018). Understanding bone cement implantation syndrome. AANA J. 86, 433–441. 31584416

[B16] Hou Y. Yuan W. (2012). Influences of disc degeneration and bone mineral density on the structural properties of lumbar end plates. Spine J. 12, 249–256. 10.1016/j.spinee.2012.01.021 22366078

[B17] Hung S. F. Liao J. C. Tsai T. T. Li Y. D. Chiu P. Y. Hsieh M. K. (2021). Comparison of outcomes between indirect decompression of oblique lumbar interbody fusion and MIS-TLIF in one single-level lumbar spondylosis. Sci. Rep. 11, 12783. 10.1038/s41598-021-92330-9 34140626 PMC8211833

[B18] Janssen I. Ryang Y. M. Gempt J. Bette S. Gerhardt J. Kirschke J. S. (2017). Risk of cement leakage and pulmonary embolism by bone cement-augmented pedicle screw fixation of the thoracolumbar spine. Spine J. 17, 837–844. 10.1016/j.spinee.2017.01.009 28108403

[B20] Karpiński R. Szabelski J. Krakowski P. Jonak J. Falkowicz K. Jojczuk M. (2023a). Effect of various admixtures on selected mechanical properties of medium viscosity bone cements: Part 3-Glassy carbon. COMPOS Struct. 343, 118307. 10.1016/j.compstruct.2024.118307

[B19] Karpiński R. Szabelski J. Krakowski P. Jonak J. Falkowicz K. Jojczuk M. (2023b). Effect of various admixtures on selected mechanical properties of medium viscosity bone cements: Part 2-Hydroxyapatite. COMPOS Struct., 118308. 10.1016/j.compstruct.2024.118308

[B21] Karpiński R. Szabelski J. Krakowski P. Jonak J. Falkowicz K. Jojczuk M. (2023c). Effect of various admixtures on selected mechanical properties of medium viscosity bone cements: Part 1-α/β tricalcium phosphate (TCP). COMPOS Struct. 343, 118306. 10.1016/j.compstruct.2024.118306

[B22] Kim D. H. Hwang R. W. Lee G. H. Joshi R. Baker K. C. Arnold P. (2020). Comparing rates of early pedicle screw loosening in posterolateral lumbar fusion with and without transforaminal lumbar interbody fusion. Spine J. 20, 1438–1445. 10.1016/j.spinee.2020.04.021 32387295

[B23] Kim H. J. Kang K. T. Chang B. S. Lee C. K. Kim J. W. Yeom J. S. (2014). Biomechanical analysis of fusion segment rigidity upon stress at both the fusion and adjacent segments: a comparison between unilateral and bilateral pedicle screw fixation. Yonsei Med. J. 55, 1386–1394. 10.3349/ymj.2014.55.5.1386 25048501 PMC4108828

[B24] Kim J. H. Ahn D. K. Shin W. S. Kim M. J. Lee H. Y. Go Y. R. (2020). Clinical effects and complications of pedicle screw augmentation with bone cement: comparison of fenestrated screw augmentation and vertebroplasty augmentation. Clin. Orthop. Surg. 12, 194–199. 10.4055/cios19127 32489541 PMC7237251

[B25] Kotheeranurak V. Jitpakdee K. Lin G. X. Mahatthanatrakul A. Singhatanadgige W. Limthongkul W. (2023). Subsidence of interbody cage following oblique lateral interbody fusion: an analysis and potential risk factors. Glob. Spine J. 13, 1981–1991. 10.1177/21925682211067210 PMC10556923 34920690

[B26] Li W. Zhu H. Hua Z. Miao D. Wang F. Tong T. (2023). Vertebral bone quality score as a predictor of pedicle screw loosening following surgery for degenerative lumbar disease. Spine (Phila Pa 1976) 48, 1635–1641. 10.1097/brs.0000000000004577 36728017 PMC10624406

[B27] Li Y. D. Hsieh M. K. Chen W. P. Lee D. M. Tsai T. T. Lai P. L. (2023). Biomechanical evaluation of pedicle screw stability after 360-degree turnback from full insertion: effects of screw shape, pilot hole profile and bone density. Front. Bioeng. Biotechnol. 11, 1151627. 10.3389/fbioe.2023.1151627 37214307 PMC10196264

[B28] Li Z. Wang X. Xie T. Pu X. Lin R. Wang L. (2023). Oblique lumbar interbody fusion combined with stress end plate augmentation and anterolateral screw fixation for degenerative lumbar spinal stenosis with osteoporosis: a matched-pair case-controlled study. Spine J. 23, 523–532. 10.1016/j.spinee.2022.12.007 36539041

[B29] Liu D. Zhang Y. Zhang B. Xie Q. y. Wang C. r. Liu J. b. (2013). Comparison of expansive pedicle screw and polymethylmethacrylate-augmented pedicle screw in osteoporotic sheep lumbar vertebrae: biomechanical and interfacial evaluations. PLoS One 8, e74827. 10.1371/journal.pone.0074827 24086381 PMC3781142

[B30] Lu T. Lu Y. (2019). Comparison of biomechanical performance among posterolateral fusion and transforaminal, extreme, and oblique lumbar interbody fusion: a finite element analysis. World Neurosurg. 129, e890–e899. 10.1016/j.wneu.2019.06.074 31226452

[B31] Ma X. Lin L. Wang J. Meng L. Zhang X. Miao J. (2023). Oblique lateral interbody fusion combined with unilateral versus bilateral posterior fixation in patients with osteoporosis. J. Orthop. Surg. Res. 18, 776. 10.1186/s13018-023-04262-x 37845750 PMC10577918

[B32] Machado G. C. Ferreira P. H. Harris I. A. Pinheiro M. B. Koes B. W. van Tulder M. (2015). Effectiveness of surgery for lumbar spinal stenosis: a systematic review and meta-analysis. PLoS One 10, e0122800. 10.1371/journal.pone.0122800 25822730 PMC4378944

[B33] Maurer M. S. Smiley D. Simsolo E. Remotti F. Bustamante A. Teruya S. (2022). Analysis of lumbar spine stenosis specimens for identification of amyloid. J. Am. Geriatr. Soc. 70, 3538–3548. 10.1111/jgs.17976 35929177 PMC9771886

[B34] Mo G. Y. Guo H. Z. Guo D. Q. Tang Y. c. Li Y. x. Yuan K. (2019). Augmented pedicle trajectory applied on the osteoporotic spine with lumbar degenerative disease: mid-term outcome. J. Orthop. Surg. Res. 14, 170. 10.1186/s13018-019-1213-y 31171020 PMC6555715

[B35] Moldovan F. (2023). Bone cement implantation syndrome: a rare disaster following cemented hip arthroplasties—clinical considerations supported by case studies. J. Pers. Med. 13, 1381. 10.3390/jpm13091381 37763149 PMC10532717

[B36] Nakajima K. Miyahara J. Nakamoto H. Kato S. Taniguchi Y. Matsubayashi Y. (2025). Correlation between severity of preoperative low back pain and postoperative outcomes in lumbar disc herniation surgery: a retrospective cohort study. Spine J. 25, 474–484. 10.1016/j.spinee.2024.10.022 39491754

[B37] Orthopaedic Rehabilitation Group of the Orthopaedic Society of the Chinese Medical Association (2025). Spinal infection group of the orthopaedic society of the Chinese medical doctor association. [Guidelines for the diagnosis and treatment of degenerative lumbar spondylolisthesis (2025 version)]. Chin. J. Orthop. 45, 261–270. 10.3760/cma.j.cn121113-20241012-00566

[B38] Park M. K. Kim K. T. Bang W. S. Cho D. C. Sung J. K. Lee Y. S. (2019). Risk factors for cage migration and cage retropulsion following transforaminal lumbar interbody fusion. Spine J. 19, 437–447. 10.1016/j.spinee.2018.08.007 30142459

[B39] Peng X. Wang X. Li Z. Xie T. Lin R. Ran L. (2024). Oblique lumbar interbody fusion combined with anterolateral screw fixation and stress endplate augmentation for treating degenerative lumbar spondylolisthesis with osteoporosis. Eur. Spine J. 33, 3467–3475. 10.1007/s00586-024-08401-8 39138674

[B40] Polikeit A. Nolte L. P. Ferguson S. J. (2003). The effect of cement augmentation on the load transfer in an osteoporotic functional spinal unit: finite-element analysis. Spine (Phila Pa 1976) 28, 991–996. 10.1097/01.brs.0000061987.71624.17 12768136

[B41] Pu X. Wang X. Ran L. Xie T. Li Z. Yang Z. (2023). Comparison of predictive performance for cage subsidence between CT-based Hounsfield units and MRI-based vertebral bone quality score following oblique lumbar interbody fusion. Eur. Radiol. 33, 8637–8644. 10.1007/s00330-023-09929-x 37462819

[B42] Rashid R. Sohrabi C. Kerwan A. Franchi T. Mathew G. Nicola M. (2024). The STROCSS 2024 guideline: strengthening the reporting of cohort, cross-sectional, and case-control studies in surgery. Int. J. Surg. 110, 3151–3165. 10.1097/js9.0000000000001268 38445501 PMC11175759

[B43] Shafiekhani P. Darabi M. Jajin E. A. Shahmohammadi M. (2023). Pedicle screw fixation with cement augmentation versus without in the treatment of spinal stenosis following posterior spinal fusion surgery, superiority according to bone mineral density: a three-arm randomized clinical trial. World Neurosurg. 180, e266–e273. 10.1016/j.wneu.2023.09.050 37741334

[B44] Silvestre C. Mac-Thiong J. M. Hilmi R. Roussouly P. (2012). Complications and morbidities of mini-open anterior retroperitoneal lumbar interbody fusion: oblique lumbar interbody fusion in 179 patients. Asian Spine J. 6, 89–97. 10.4184/asj.2012.6.2.89 22708012 PMC3372554

[B45] Sin D. A. Heo D. H. (2019). Comparative finite element analysis of lumbar cortical screws and pedicle screws in transforaminal and posterior lumbar interbody fusion. Neurospine 16, 298–304. 10.14245/ns.1836030.015 31154694 PMC6603848

[B46] Tandon V. Kalidindi K. K. V. Pacha S. Bhat M. R. (2020). A prospective study on the feasibility, safety, and efficacy of a modified technique to augment the strength of pedicle screw in osteoporotic spine fixation. Asian Spine J. 14, 357–363. 10.31616/asj.2019.0211 31906610 PMC7280929

[B47] Tsagkaris C. Calek A. K. Fasser M. R. Spirig J. M. Caprara S. Farshad M. (2023). Bone density optimized pedicle screw insertion. Front. Bioeng. Biotechnol. 11, 1270522. 10.3389/fbioe.2023.1270522 37954015 PMC10639121

[B48] Vaishya R. Chauhan M. Vaish A. (2013). Bone cement. J. Clin. Orthop. Trauma. 4, 157–163. 10.1016/j.jcot.2013.11.005 26403875 PMC3880950

[B49] Zhao L. Xie T. Wang X. Yang Z. Pu X. Lu Y. (2022). Clinical and radiological evaluation of cage subsidence following oblique lumbar interbody fusion combined with anterolateral fixation. BMC Musculoskelet. Disord. 23, 214. 10.1186/s12891-022-05165-4 35248042 PMC8898418

[B50] Zhong Y. Wang Y. Zhou H. Gan Z. Qu Y. Hua R. (2023). Biomechanical study of two-level oblique lumbar interbody fusion with different types of lateral instrumentation: a finite element analysis. Front. Med. (Lausanne) 10, 1183683. 10.3389/fmed.2023.1183683 37457575 PMC10345158

